# Supplementary material for: Synovial fluid proteome profile of surgical versus chemical induced osteoarthritis in rabbits
Source: PeerJ. 2022 Feb 23;10:e12897. doi: 10.7717/peerj.12897 (PMC8881915; doi:10.7717/peerj.12897)
Supplement: Supplemental Information 1 — Spot quantification for proteins were analyzed by Progenesis SameSpot software. Spot volume normalization and calculation are performed by the software automatically. [file peerj-10-12897-s001.docx]

**Supplementary figures for Table 1.** Spot quantification for proteins were analyzed by Progenesis SameSpot software. Spot volume normalization and calculation are performed by the software automatically.

**Alpha-1-antiproteinase F precursor**


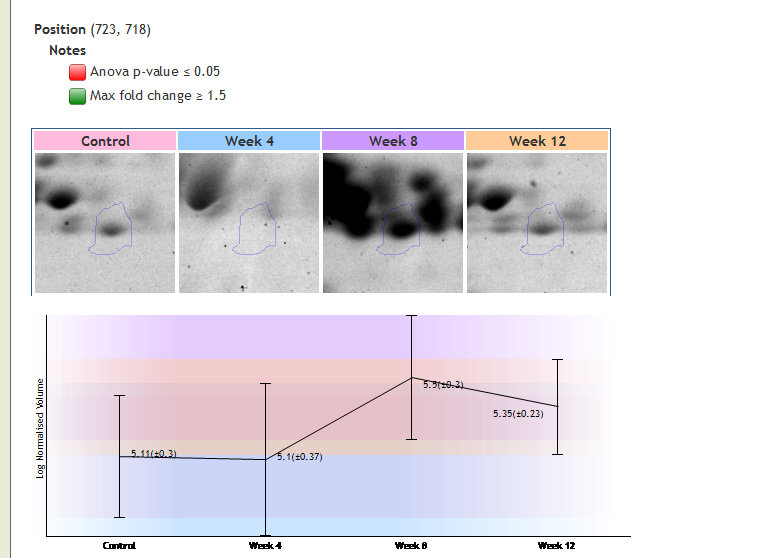


**Histidine-rich glycoprotein**


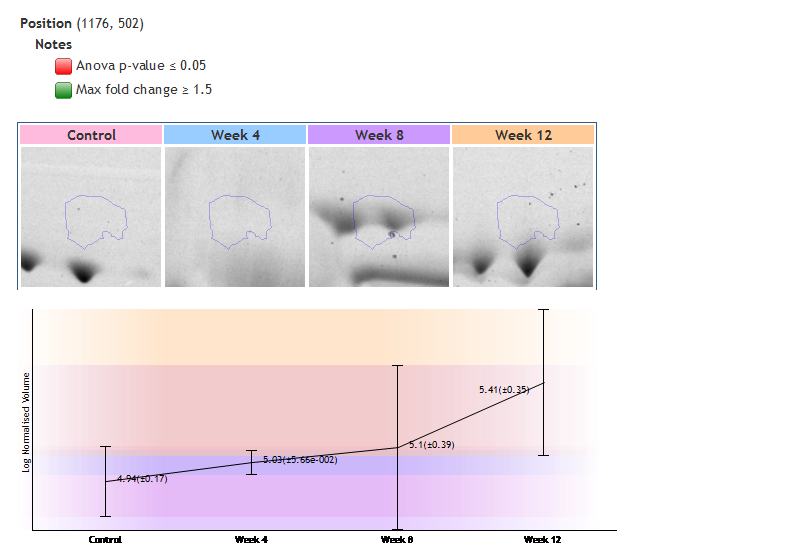


**Retinol-binding protein 4**


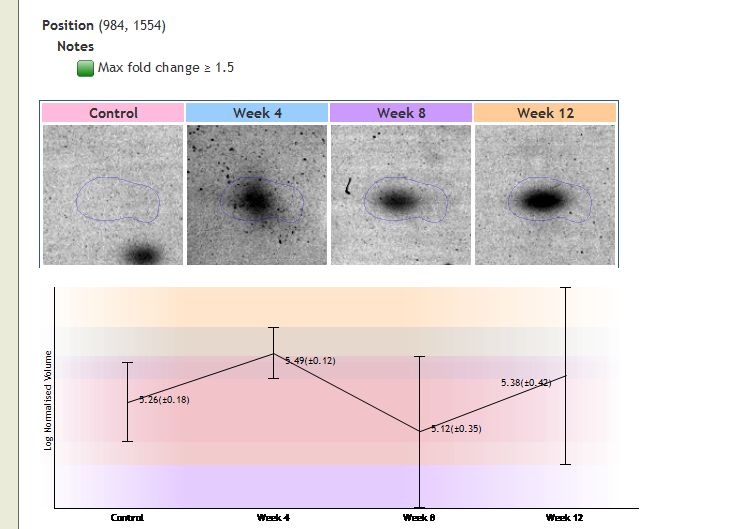


**Beta-actin-like protein 2 isoform X1**


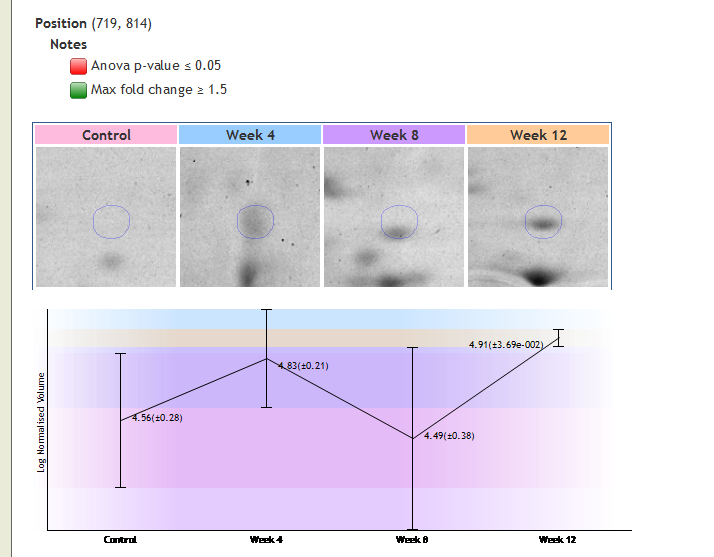


**Full=Ig kappa-b4 chain C region**


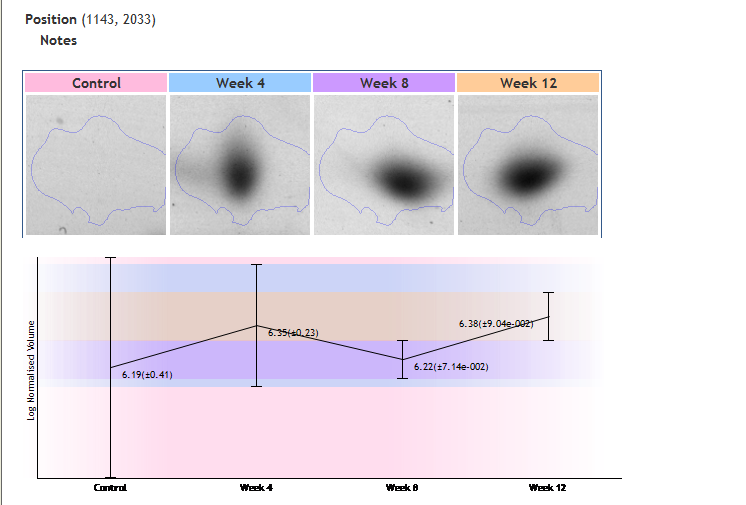


**Serotransferrin**


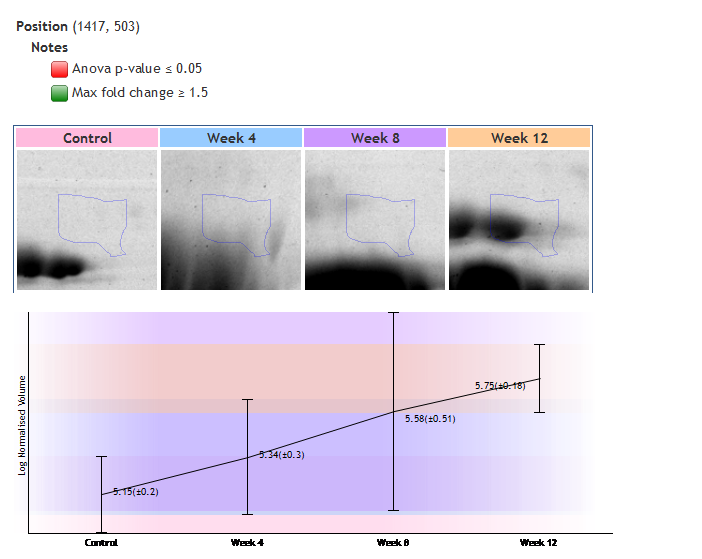


**Gelsolin isoform**


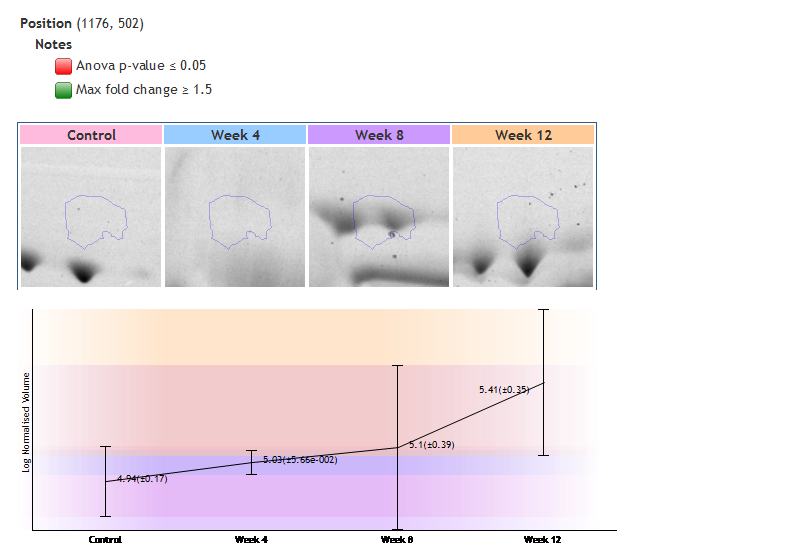


**Serpin peptidase inhibitor**

**
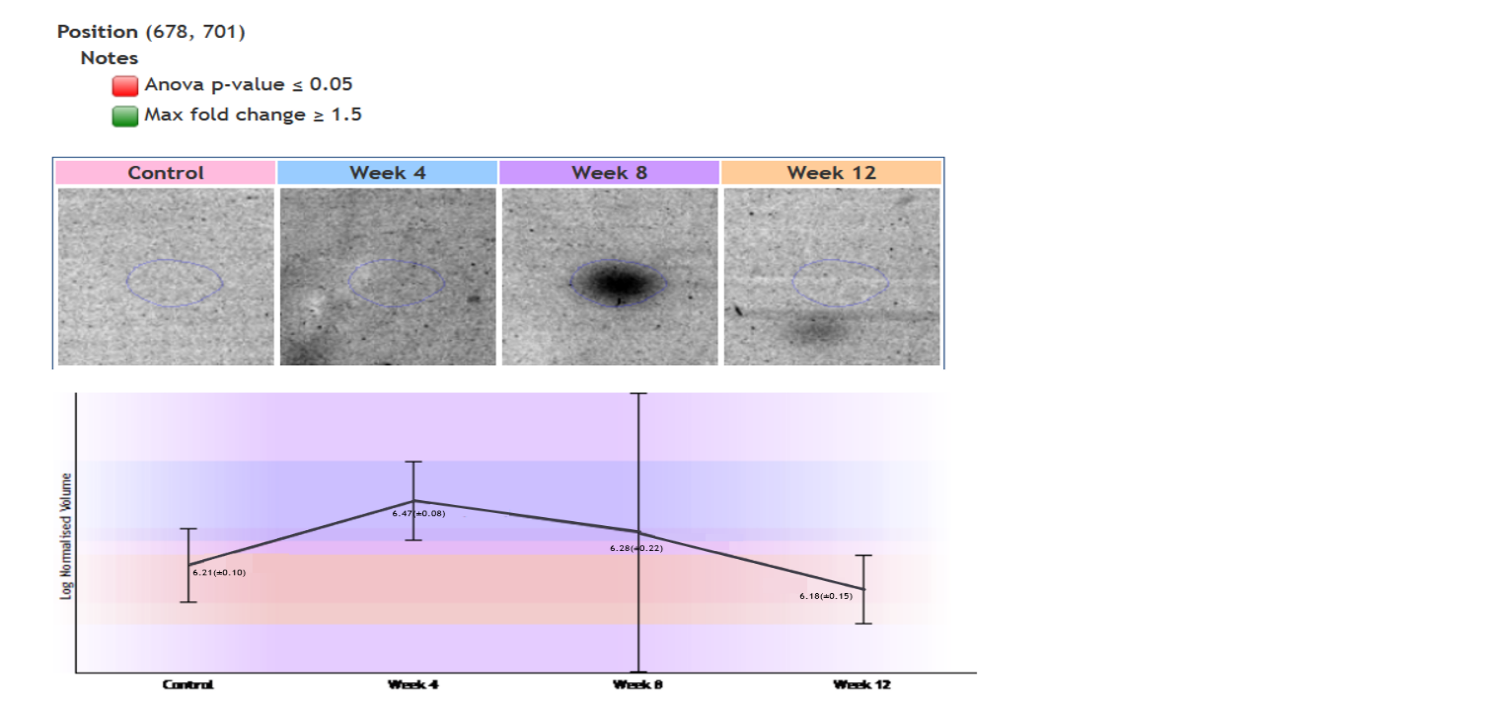
**

**Haptoglobin precursor**

**
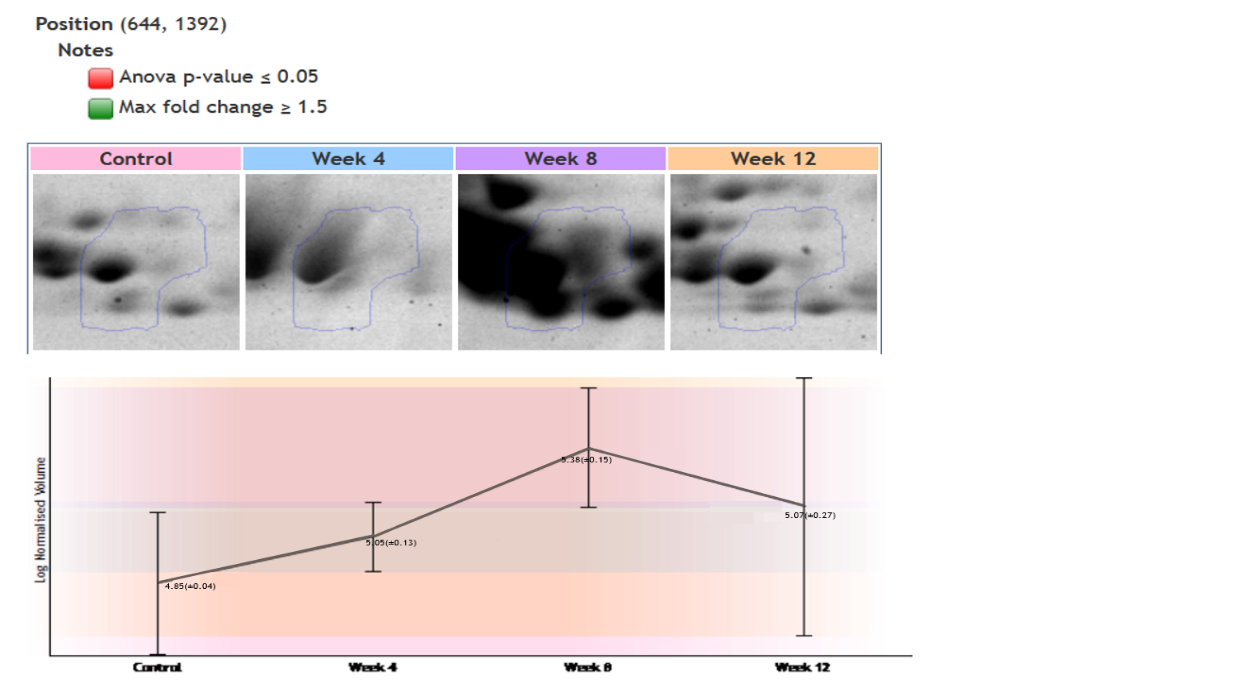
**

**Apolipoprotein I-IV precursor**

**
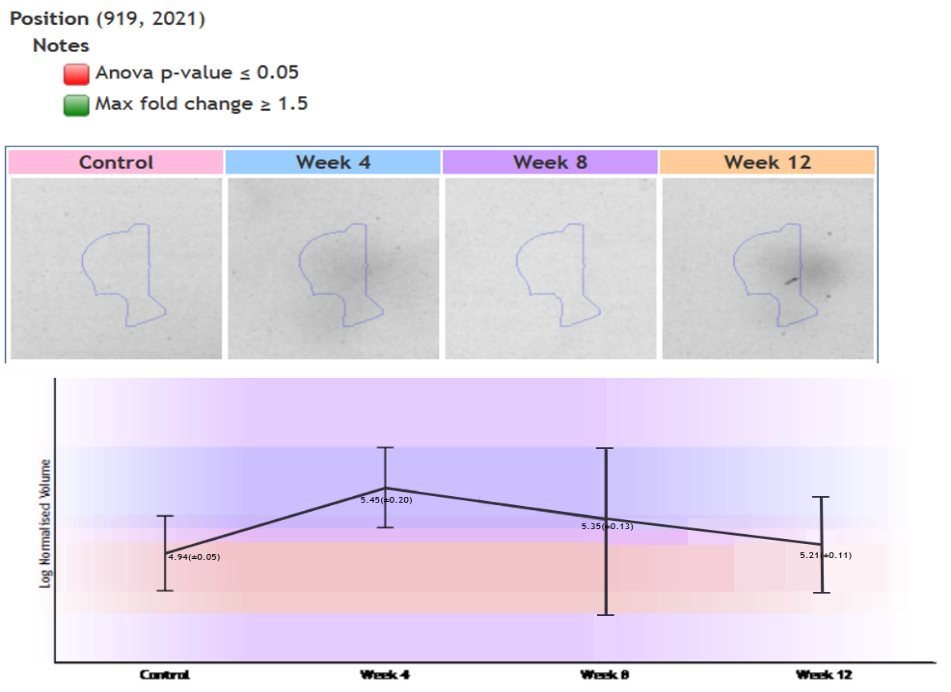
**
